# Higher CD4/CD8 ratio of pleural effusion predicts better survival for lung cancer patients receiving immune checkpoint inhibitors

**DOI:** 10.1038/s41598-021-89043-4

**Published:** 2021-04-30

**Authors:** Po-Hsin Lee, Tsung-Ying Yang, Kun-Chieh Chen, Yen-Hsiang Huang, Jeng-Sen Tseng, Kuo-Hsuan Hsu, Yu-Chen Wu, Ko-Jiunn Liu, Gee-Chen Chang

**Affiliations:** 1 Division of Chest Medicine, Department of Internal Medicine, Taichung Veterans General Hospital, No.1650, Sect. 4, Taiwan Boulevard, Taichung, 407 Taiwan; 2 Faculty of Medicine, School of Medicine, National Yang-Ming University, Taipei, Taiwan; 3 Division of Pulmonary Medicine, Department of Internal Medicine, Chung Shan Medical University Hospital, No. 110, Sec. 1, Jianguo N. Rd., South Dist., Taichung City, 402 Taiwan, ROC; 4 Institute of Medicine, Chung Shan Medical University, Taichung, Taiwan; 5 School of Medicine, Chung Shan Medical University, Taichung, Taiwan; 6 Institute of Biomedical Sciences, National Chung Hsing University, Taichung, Taiwan; 7 Division of Critical Care and Respiratory Therapy, Department of Internal Medicine, Taichung Veterans General Hospital, No.1650, Sect. 4, Taiwan Boulevard, Taichung, 407 Taiwan; 8 National Institute of Cancer Research, National Health Research Institutes, 2F, No.367, Sheng-Li Road, Tainan, 704 Taiwan; 9 Graduate Institute of Medicine, College of Medicine, Kaohsiung Medical University, Kaohsiung, Taiwan; 10 Institute of Clinical Pharmacy and Pharmaceutical Sciences and Institute of Clinical Medicine, National Cheng Kung University, Tainan, Taiwan; 11 School of Medical Laboratory Science and Biotechnology, Taipei Medical University, Taipei, Taiwan

**Keywords:** Non-small-cell lung cancer, Cancer immunotherapy, Tumour immunology

## Abstract

Pleural effusion is a rare immune-related adverse event for lung cancer patients receiving immune checkpoint inhibitors (ICIs). We enrolled 281 lung cancer patients treated with ICIs and 17 were analyzed. We categorized the formation of pleural effusion into 3 patterns: type 1, rapid and massive; type 2, slow and indolent; and type 3, with disease progression. CD4/CD8 ratio of 1.93 was selected as the cutoff threshold to predict survival. Most patients of types 1 and 2 effusions possessed pleural effusion with CD4/CD8 ratios ≥ 1.93. The median OS time in type 1, 2, and 3 patients were not reached, 24.8, and 2.6 months, respectively. The median PFS time in type 1, 2, and 3 patients were 35.5, 30.2, and 1.4 months, respectively. The median OS for the group with pleural effusion CD4/CD8 ≥ 1.93 and < 1.93 were not reached and 2.6 months. The median PFS of those with pleural effusion CD4/CD8 ≥ 1.93 and < 1.93 were 18.4 and 1.2 months. In conclusion, patients with type 1 and 2 effusion patterns had better survival than those with type 3. Type 1 might be interpreted as pseudoprogression of malignant pleural effusion. CD4/CD8 ratio ≥ 1.93 in pleural effusion is a good predicting factor for PFS.

## Introduction

Immune checkpoint inhibitors (ICIs) have become promising agents against a variety of cancers. However, in some patients, concomitant immune-related adverse events (irAEs) develop. Among organs affected byICI treatment, pleural involvement is rare. Under ICI treatment, pseudoprogression may develop, with a transient increase in the tumor size before regression^1^. Pseudoprogression in lung-cancer patients occurs not only in the solid part of the tumor but also has been reported in malignant spread to pleural and pericardial space with the presentation of rapidly accumulating recurrent effusions^2^. The clinical course and outcomes of patients receiving ICIs followed by pleural effusion development are poorly known.

IrAEs involving different organs may result from various mechanisms^3^. For example, in myocarditis, the inflammatory infiltration of T cells is predominantly CD8^4^, whereas in pericardial involvement, T cell infiltration is predominantly CD4^5^. Which types of lymphocytes are involved in pleural effusion under ICIs remained unknown.

In the present study, we aimed to categorize the clinical presentations of ICI-related pleural effusion and analyze the lymphocyte components in the pleural effusion in relation to the clinical outcomes of non-small cell lung cancer (NSCLC) patients receiving ICIs.

## Methods

### Study design

NSCLC patients were enrolled retrospectively at Taichung Veterans General Hospital from Oct 2015 to Dec 2019, during which ICI treatments were initiated. The last follow-up was on May 31, 2020. Eligible patients all had non-infectious pleural effusion after ICI use. The exclusion criteria were as follows: no cytology results of pleural effusion, mortality of unknown etiology, specimens for lymphocyte analysis not from pleural effusion, the duration from the last dose of ICI to development of pleural effusion exceeded 12 months. Patients receiving ICIs and docetaxel were excluded since docetaxel was known to cause pleural effusion^6^. Patients had (1) chest trauma prior to, or during, ICI treatment, (2) invasive procedures directed into the pleural cavity prior to, or during ICI treatment (with the exception of a pleural catheter and pleural biopsy), (3) talc pleurodesis were not included because all of these interventions would likely affect lymphocyte measurements in the PE. This study was approved by the Institutional Review Board of Taichung Veterans General Hospital (IRB No. CF16018A). Written informed consents for clinical data records, genetic and immunological testing were obtained from all patients. All methods were carried out in accordance with the relevant approved guidelines and regulations.

### Definition of disease progression and development of pleural effusion

We categorized the pattern of pleural effusion formation of our patients into three types: (1) Type1: rapid production, without disease progression, within one month after ICIs use, malignant pleural effusion was recorded after ICI treatment which turns from positive to negative from serial cytology exams (2) Type 2: slow production, without disease progression, usually one month after ICIs use, (3) Type 3: pleural effusion due to disease progression even with ICI treatments. Disease progression was defined as follows: (1) newly developed malignant pleural effusion which did not turn from positive to negative from serial cytology exams, (2) pleural effusion negative for malignancy but with disease progression at other locations.

We defined newly developed pleural effusion as follows: (1) no pleural effusion before ICI use, but effusion developed after treatments, (2) pleural effusion existed before ICI use and rate of effusion accelerated after treatments. The definition of acceleration was as follows: (1) a pigtail catheter was inserted for symptomatic relief of pleural effusion, or (2) the frequency of thoracentesis increased (eg. from once a month to once a week).

### Pleural effusion analysis and lymphocyte subset measurement

We analyzed lymphocyte subsets in pleural effusion which was collected the first time patients had received thoracentesis after ICI use. Since no less than 150 ml of pleural effusion was required for analysis, the insufficient pleural effusions of some patients were not analyzed at the first time.

Mononuclear cells in the pleural effusion were collected through density gradient centrifugation with Ficoll‐Paque. The analysis of immune cells in pleural effusion was performed with fresh samples. For cell-type analyses based on surface molecules, cells were first stained with different fluorescence‐labeled monoclonal antibodies and then analyzed with flow cytometry. Cells were gated based on the forward scatter channel and side scatter channel to select lymphocytes. For the T cell subset study, cells were stained with phycoerythrin (PE)‐anti-CD3, PerCP‐anti-CD4, and FITC‐anti-CD8, and cells expressing CD3 were gated for CD4 and CD8 analyses. For the B cell study, cells were stained with FITC-anti-CD3 and PE-anti-CD19, and the percentage of CD3−CD19+ cells was determined. Isotype‐matched control monoclonal antibodies were obtained from BD PharMingen and BioLenged. The laser set up of the flow cytometer was determined based on the fluorescent intensity of each sample stained with the isotype-control antibodies and the results were analyzed with the FlowJo software, v7.6.2 (BD). Details of equipment and antibody are shown in online supplemental Table 1.

### Analyses of cytokine productions in pleural effusion

Pleural effusion was first centrifuged to remove cells and debris. The supernatant was collected and stored at − 80 °C. The supernatant derived from pleural effusion of different patients was thawed at the same time for cytokine analysis. We used the sandwich‐enzyme‐linked immunosorbent assay (ELISA) with the OptEIA kit (BD Pharmingen) to detect levels of IL-1, IL-2, IL-10, IL-12p70, and IFN-γ in the pleural effusion. We also used the DuoSet ELISA kit (R&D Systems Inc., Minneapolis, MN) to detect levels of IL-8 and IL-17. IL-6 and TNF-α levels were detected by ELISA (Invitrogen, Thermo Fisher Scientific, Waltham MA). Detection ranges with ELISA are shown in online supplemental Table 2. The samples were diluted for the ELISA assay if necessary.

### Identification of driver mutations and PD-L1 assay

Tumor specimens were procured for oncogenic mutation analyses as previously reported^7^. Five oncogenic drivers, including *EGFR*, *KRAS*, *BRAF*, *HER2*, and *EML4-ALK*, were tested. For patients with squamous cell carcinoma, oncogenic mutation analyses were not routinely performed.

Three commercial Programmed Death-ligand 1 (PD-L1) IHC assays, 22C3, SP142, and SP263, were performed for all patients when adequate specimens were available. The PD-L1 IHC 22C3 pharmDx was conducted on the DAKO Autostainer Link 48, while the Ventana PD-L1 SP142 and SP263 assays were conducted on the Ventana BenchMark platform.

### Data records and response evaluation

Clinical data of individual patients included age, gender, Eastern Cooperative Oncology Group Performance Status (ECOG PS), tumor stage, smoking status, and thyroid function. The age, ECOG PS, and tumor stage were evaluated while ICIs were initiated. The overall survival (OS) and progression-free survival (PFS) were analyzed from the beginning of ICI treatment. TNM (tumor, node, and metastases) staging was performed according to the 8th edition of the American Joint Committee for Cancer (AJCC) staging system. We adopted here unidimensional measurements as defined by Response Evaluation Criteria in Solid Tumors (RECIST) version 1.1.

### Statistical methods

Fisher's exact test and Mann–Whitney U test were used to compare intergroup differences for categorical and continuous variables as appropriate. Univariate and multivariate Cox proportional hazard regression models were used to estimate the hazard ratio. The OS and PFS were estimated using the Kaplan–Meier method, whereas the between-group differences were assessed using the stratified log-rank test. Two-tailed tests with p values < 0.05 were considered statistically significant. Receiver operating curves analysis was applied to identify the optimal cutoff threshold of pleural effusion CD4/CD8 and B cell ratios in predicting survival.

All analyses were performed with the IBM SPSS Statistics package, version 23 (IBM Corporation, Armonk, NY).

## Results

### Patient characteristics

**Figure 1:**
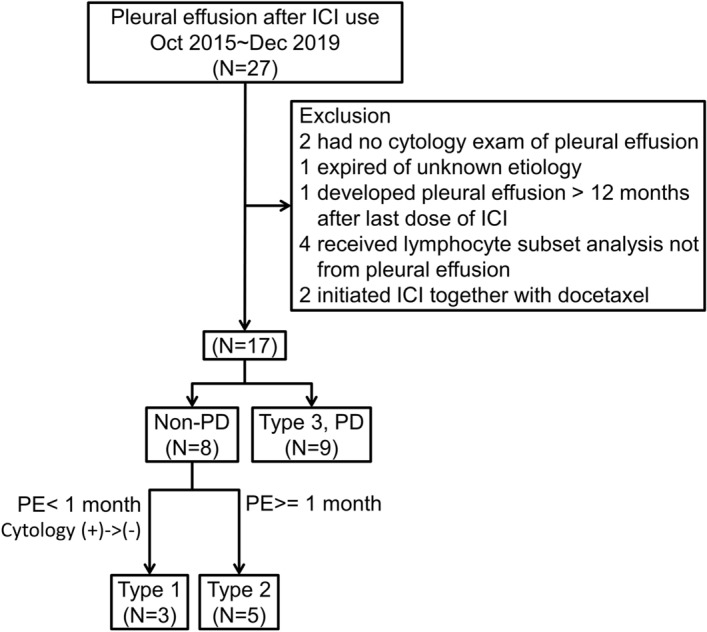
Algorithm for enrollment and follow-up of the study participants. Type 1: rapid growth of pleural effusion within 1 month after immune checkpoint inhibitor use. Malignant cells were found in pleural effusion after the initial treatment but were absent in the following serial thoracenteses. Type 2: slow growth of pleural effusion developed more than one month after immune checkpoint inhibitor use. Type 3: pleural effusion due to disease progression. ICI: immune checkpoint inhibitor, PD: disease progression, PE: pleural effusion.

We included a total of 281 advanced (stage IIIB/IV) NSCLC patients with ICIs initiated. Among these patients, 168 patients were treated with pembrolizumab, 43 with nivolumab, 47 with atezolizumab, and 23 with durvaluamb. Among them, 27 developed pleural effusion after ICI use, with 10 excluded. Among the remaining 17 patients, three were categorized as type 1, 5 as type 2, and 9 as type 3 (Fig. 1).

**Figure 2:**
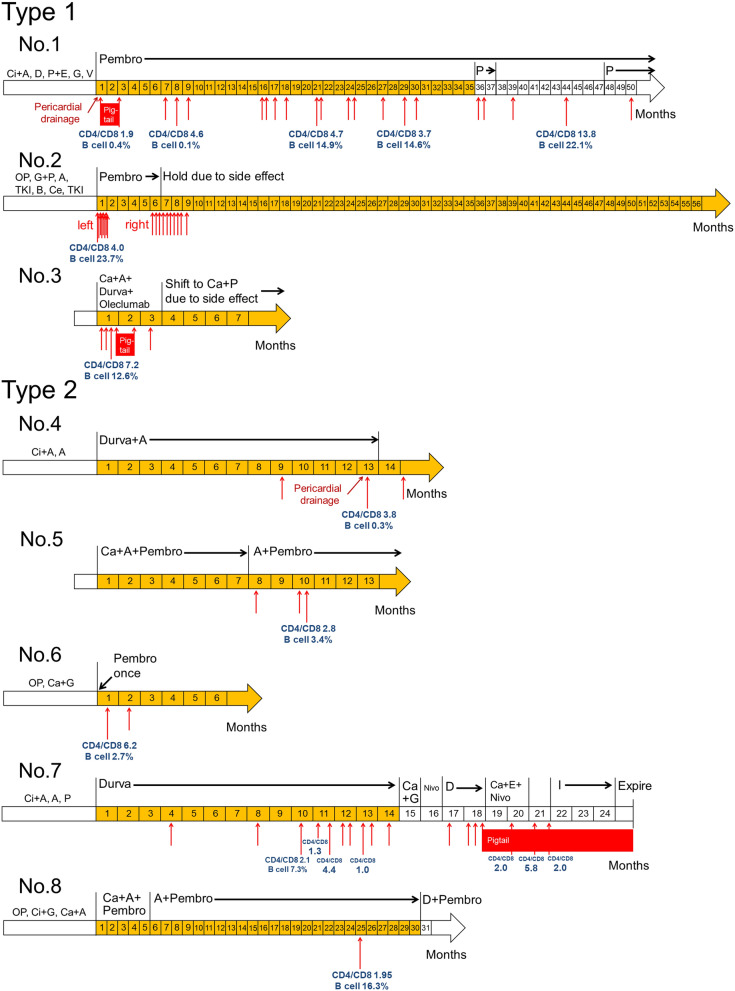
Disease course and treatment timeline for patients with type 1 and type 2 pleural effusions. Red Arrow: Pleural effusion requiring thoracentesis. Time line highlighted in yellow: progression free survival (the number within the timeline indicates the month after the immune checkpoint inhibitor (ICI) initiation). The left side of the yellow bar refers to the initiation of the ICI. The regimens of each patient were listed above the timeline. Ci, cisplatin; A, pemetrexed (Alimta); D, docetaxel; P, paclitaxel; E, etoposide; G, gemcitabine; V, vinorelbine; Pembro, pembrolizumb; OP, surgical intervention; TKI, *EGFR*-tyrosine kinase inhibitor; B, bevacizumab; Ce, cetuximab; Ca, carboplatin; Durva, durvalumab; Nivo, nivolumab; I, irinotecan.

Their descriptive characteristics are summarized in Table 1. All the patients had reached advanced stages of lung cancer before ICI use. Adenocarcinoma was diagnosed in 13 patients, and three of them harboring *EGFR* mutations. Negative PD-L1 expression was found in 6 patients, low PD-L1 expression in 4 patients, and high PD-L1 expression in 5 patients. Of the 17 patients, 13 received lymphocyte subset analyses of their pleural effusions within one month after the first thoracentesis. Clinical data and outcomes of the enrolled patients are shown in Table 2. The disease course and treatment timeline for patients of types 1 and 2 are shown in Fig. 2. Supplemental Figure 1 illustrated radiological change for each patient with measurable lesion compared with baseline diameter.

**Table 1:** Demographic data and characteristics of different pleural effusion types.

	All (N = 17)	Type 1 + type 2 (N = 8)	Type 3 (N = 9)	*p* value^a^
Age, medium (IQR)	60.1 (52.6–65.8)	63.0 (52.4–68.6)	58.9 (53.9–61.6)	0.423
**Gender, N (%)**				0.335
Male	10 (58.8)	6 (75)	4 (44.4)	
Female	7 (41.2)	2 (25)	5 (55.6)	
**Smoking status, N (%)**				0.347
Ever smoker	8 (47.1)	5 (62.5)	3 (33.3)	
Never smoker	9 (52.9)	3 (37.5)	6 (66.7)	
**Stage, N (%)**				1.000
IIIB–IIIC	2 (11.8)	1 (12.5)	1 (11.1)	
IVA–IVB	15 (88.2)	7 (87.5)	8 (88.9)	
**Brain metastasis before ICI, N (%)**				0.620
Yes	5 (29.4)	3 (37.5)	2 (22.2)	
No	12 (70.6)	5 (62.5)	7 (77.8)	
**ECOG PS, N (%)**				0.206
0–2	14 (82.4)	8 (100)	6 (66.7)	
3–4	3 (17.6)	0 (0)	3 (33.3)	
**Pathology and driver mutation, N (%)**				1.000
ADC without driver mutation	10 (58.8)	5 (62.5)	5 (55.6)	
ADC with *EGFR* mutation	3 (17.6)	1 (12.5)	2 (22.2)	
Non-ADC NSCLC	4 (23.5)	2 (25)	2 (22.2)	
**PD-L1, N (%)**				0.147
< 1%	6 (35.3)	1 (12.5)	5 (55.6)	
1–49%	4 (23.5)	2 (25)	2 (22.2)	
≥ 50%	5 (29.4)	4 (50)	1 (11.1)	
N/A	2 (11.8)	1(12.5)	1(11.1)	
**ICI type, N (%)**				0.689
Pembrolizumab	10 (58.8)	4 (50)	6 (66.7)	
Nivolumab	1 (5.9)	1 (12.5)	0 (0)	
Atezolizumab	1 (5.9)	0 (0)	1 (11.1)	
Durvalumab	5 (29.4)	3 (37.5)	2 (22.2)	
**Hypothyroidism after ICI use, N (%)**				0.315
Yes	6 (35.3)	2 (25)	4 (44.4)	
No	9 (52.9)	6 (75)	3 (33.3)	
N/A	2 (11.8)		2 (22.2)	
**Pericardial effusion requiring drainage after ICI, N (%)**				0.576
Yes	3 (17.6)	2 (25)	1 (11.1)	
No	14 (82.4)	6 (75)	8 (88.9)	
Interval from ICI to 1st thoracentesis, months, medium (IQR)	0.63 (0.30–4.87)	1.9 (0.3–6.9)	0.6 (0.3–1.9)	0.815
Interval from 1st thoracentesis to CD4/CD8 ratio, months, medium (IQR)	0 (0–0.97)	0.9 (0–2.7)	0 (0–0)	0.093
**CD4/CD8 ratio, N (%)**				0.036
≥ 1.93	10 (58.8)	7 (87.5)	3 (33.3)	
< 1.93	7 (41.2)	1 (12.5)	6 (66.7)	
**B cell ratio, N (%)**				0.131
≥ 6.09	5 (29.4)	4 (50)	1 (11.1)	
< 6.09	12 (70.6)	4 (50)	8 (88.9)	

ICI, immune checkpoint inhibitor; ECOG PS, Eastern Cooperative Oncology Group performance status; ADC, adenocarcinoma; NSCLC, non-small cell lung cancer; N/A, not applicable; PR, partial response; SD, stable disease; PD, disease progression.

^a^Probability value by Mann–Whitney U test and Fisher's exact test.

**Table 2:** Clinical data and outcomes of lung cancer patients developing pleural effusion after ICI use.

No	1	2	3	4	5	6	7	8	9	10	11	12	13	14	15	16	17
Age	60.1	79	65.8	47.6	74.3	66.6	51.9	52.6	61.6	56.4	64.4	60.3	45.7	47.6	58.9	68.1	53.9
Gender	M	F	M	F	M	M	M	M	F	M	M	F	F	M	M	F	F
Smoking	E	N	E	N	N	E	E	E	N	E	N	N	N	N	N	E	E
PS	2	2	1	1	1	2	1	1	1	3	2	4	1	1	3	1	1
Cell type/*EGFR*	ADC	ADC	ADC	IMA	ADC^@1^	SqCC	ADC	ADC	ADC	ADC	SqCC	ADC^@2^	ADC	ADC	ADC^@1^	ADC	SqCC
Stage	IVB	IVA	IVA	IIIC	IVB	IVB	IVA	IVB	IVB	IVB	IVB	IVB	IVA	IVB	IVB	IVB	IIIB
Brain mets	No	No	No	No	Yes	Yes	No	Yes	No	No	No	No	No	Yes	Yes	No	No
PD-L1	(−)	High (+)	Low (+)	N/A	High (+)	High (+)	High (+)	Low (+)	Low (+)	(−)	(−)	High (+)	(−)	(−)	N/A	(−)	Low (+)
Previous Tx to ICI (m)	0.7	5.6	1st line	4.1	1st line	8.4	0.9	1.2	0.1	1.2	1st line	0.4	1st line	1.0	0.9	1st line	1.2
ICI	n	P	D	D	P	P	D	P	P	P	A	P	P	P	P	D	D
Cycle	92^$^	8	4	15	14^$^	1	26	26^$^	1	1	2	5	6	2	1	6	39^$^
PFS (m)	35.5	56.6*	7.8*	14.5*	13.3*	6.6*	14	30.2	0.5	0.9	1.6	3.2	5	1.2	0.8	1.4	18.4
OS (m)	50.5^#^	56.6^#^	7.8^#^	14.5^#^	13.3^#^	6.6^#^	24.8	31.3^#^	0.6	2.2	2.3	4.7	29.9^#^	2.6	0.8	14	20.9^#^
ICI to PE (m)	0.2	0.3	0.3	7.9	6.5	0.3	3.5	25	0.2	0.3	0.6	0.2	4.9	0.7	0.6	1.9	19.1
PE to CD4/CD8 (m)	1.7	0	0.1	4.5	2.3	0	5.9	0	0	0	1	0	0	0.7	0	0	0
Volume of thoracentesis (ml)	N/A	1050	830	N/A	600	200	960	130	460	400	440	510	180	400	860	680	790
TNC/lym ratio (per μl/%)	N/A	1109/51%	3652/21%	178/16%	614/73%	2230/74%	311/20%	2127/63%	12/33%	1754/89%	293/22%	777/61%	2198/54%	194/95%	2553/9%	5066/59%	1584/51%
Initial CD4/CD8	1.89	4.01	7.22	3.84	2.83	6.22	2.1	1.95	1.07	1.85	13.23	4.78	1.36	1.9	1.9	1.9	8.5
Initial B cell ratio (%)	0.3	23.7	12.6	0.3	3.4	2.7	7.3	16.3	2.7	1.6	1.5	4.9	0.8	3.8	2.5	4.5	34
PE before ICI	Yes	Yes	Yes	No	No	Yes	Yes	No	No	Yes	Yes	Yes	Yes	Yes	Yes	Yes	Yes
PE cytology before ICI	N/A	N/A	(+)	N/A	N/A	(−)	N/A	N/A	N/A	N/A	(−)	(−)	(+)	(+)	(+)	N/A	N/A
1^st^ PE after ICI cytology	(+)	(+)	(+)	(−)	(−)	(−)	(−)	(−)	(−)	(−)	(−)	(+)	(+)	(−)	(+)	(+)	(+)
Serial PE cytology	(+) → (−)	(+) → (−)	(+) → (−)	(−) → (−)	(−) → (−)	(−) → (−)	(−) → (−)^&^	PE once	PE once	(−) → (−)	(−) → (−)	(+) → (+)	(+) → (+)	(−) → (+)	(+) → (+)	(+) → (+)	(+) → (+)
Pleural effusion type	Type 1	Type 1	Type 1	Type 2	Type 2	Type 2	Type 2	Type 2	Type 3	Type 3	Type 3	Type 3	Type 3	Type 3	Type 3	Type 3	Type 3

ICI, immune checkpoint inhibitor; M, male; F, female; E, ever smoker; N, never smoker; PS, Eastern Cooperative Oncology Group performance status; ADC, adenocarcinoma; IMA, invasive mucinous adenocarcinoma; @1, *EGFR* L858R mutation; SqCC, squamous cell carcinoma; @2, *EGFR* G719S mutation; Brain mets, brain metastasis before ICI use; Previous Tx to ICI: the time between previous treatment and the start of ICI use; n, nivolumab; P, pembrolizumab; D, durvalumab; A, atezolizumab; $, ongoing ICI use; m, month; *, no disease progression; #, survive; ICI to PE, time from ICI use to 1st thoracentesis; PE, pleural effusion; PE to CD4/CD8; interval from 1st thoracentesis to pleural effusion lymphocyte subset analysis; TNC, total nucleated cell count in the pleural effusion; PE before ICI, pleural effusion noted by image before ICI use; cytology (+), positive for malignant cell; cytology (−), negative for malignant cell; N/A, not applicable; &, the effusion was positive for malignancy for 2 times but most times it showed negative for malignancy; PE once, the patient received thoracentesis for only one time.

### Pleural effusion with CD4/CD8 ratio ≥ 1.93 is a good predictor for survival

Cell surface expressions of CD4 and CD8 on T cells are shown in online supplemental Figure 2. Patient no.1 and no.7 received serial pleural effusion CD4/CD8 ratios examination and the former had progressively elevated ratios (Fig. 2 and online supplemental Figure 2 Patient no.1). The initial CD4 and CD8 ratios as a proportion of cells expressing CD3 of each patient are illustrated in online supplemental Figure 3A,B. The initial CD4/CD8 ratios are shown in online supplemental Figure 3C. Receiver operating curves analysis was applied to identify the optimal cutoff threshold (online supplemental Figure 4). PFS of 3.7 months and OS of 12.0 months were used as cut-points according to median survivals in KEYNOTE-001 trial^8^. The reasons for choosing the survival times from the trial are as follows: The population in our study was highly heterogeneous. They expressed different PD-L1 levels, may or may not harbor driving mutations, had various histories of previous treatment before ICI initiation. KEYNOTE-001 trial was a phase I study designed to assess the efficacy and safety of pembrolizumab with a relatively relaxed criterion of enrollment. The inclusion criteria in the later trials were relatively strict. In KEYNOTE-024 trial, patients were enrolled if previously untreated, with PD-L1 expression ≥ 50%, and harboring no *EGFR* or *ALK* mutation^9^. In KEYNOTE-042 trial, the inclusion criterion was similar besides the PD-L1 expression status (at least 1%)^10^. As for another ICI, there was not much difference between the median survival times of atezolizumab and pembrolizumab in the phase I studies^11^. Because most patients in our study received pembrolizumab and the population and inclusion criterion of KEYNOTE-001 trial were most similar to that of our study, we choose the median survival times from the trial to set the cut-points. In predicting OS, two patients were excluded from analysis because the follow-up time failed to last > 12.0 months after ICI use. The cutoff threshold was set at 1.93 in predicting OS, PFS, and the type of pleural effusion. The CD4/CD8 ratios divided by type 1/2 and type 3 were provided as a scatter plot in online supplemental Figure 3E.

**Figure 3:**
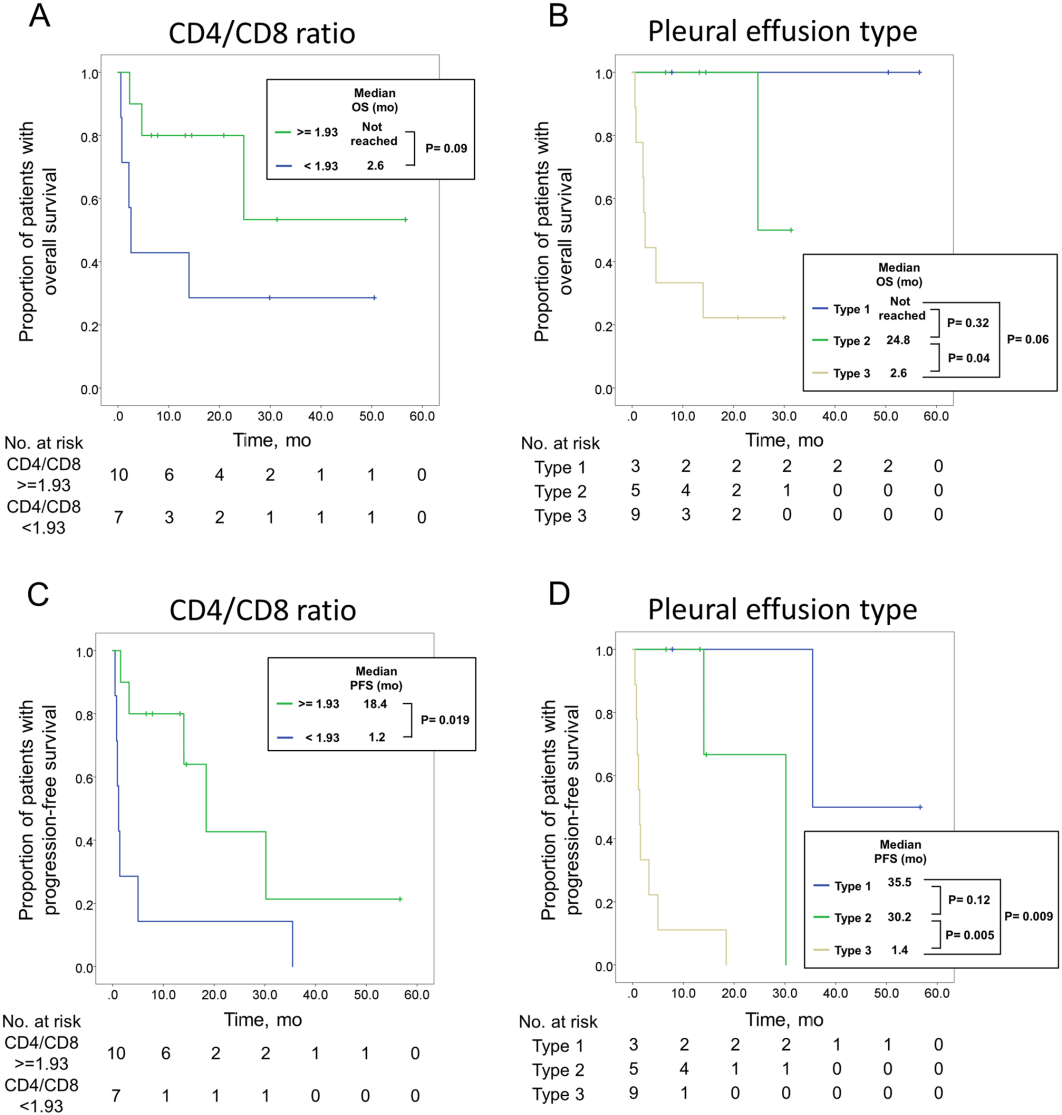
Overall survival (OS) and progression free survival (PFS) according to pleural effusion CD4/CD8 ratios and types. (**A**) OS and (**C**) PFS shown by Kaplan–meier methods according to their different CD4/CD8 ratios in pleural effusion. (**B**) OS and (**D**) PFS plotted by Kaplan–meier methods according to types of pleural effusion.

In univariate and multivariate analyses for OS or PFS before ICI use, Cox-regression showed no significantly related risk factors, like age, gender, smoking history, PD-L1 status, and brain metastasis (Tables 3 and 4). ECOG PS was identified as significant predictors associated with OS in both univariate and multivariate analyses Similarly, ECOG PS and CD4/CD8 ratio were predictors of PFS. High pleural effusion CD4/CD8 ratios correlating with longer PFS and OS (Fig. 3) was more commonly found in patients of types 1 and 2 (online supplemental Table 3). High CD4/CD8 ratios also correlated with PD-L1 expression status (online supplemental Table 3). The median OS in CD4/CD8 ≥ 1.93 group was not reached, whereas the median OS in CD4/CD8 < 1.93 was 2.6 months (Fig. 3A). The median PFS of patients with CD4/CD8 ≥ 1.93 was 18.2 months, and with this ratio < 1.93 was 1.2 months (Fig. 3C).

**Table 3:** Univariate and multivariate analyses of risk factors for overall survival.

Variable	Univariate		Multivariate	
	HR (95% CI)	*p* value^a^	HR (95% CI)	*p* value^a^
Age	1.00 (0.93–1.07)	0.866		
Male gender	1.38 (0.32–5.85)	0.666		
Never smoker	2.00 (0.48–8.43)	0.344		
**ECOG PS**				
0–2	1			
3–4	7.81 (1.52–40.29)	0.014	8.82 (1.48–52.66)	0.017
Brain mets before ICI	0.97 (0.19–4.89)	0.973		
**PD-L1**				
< 1%	2.01 (0.36–11.11)	0.213		
1–49%	0.77 (0.07–8.50)	0.829		
≥ 50%	1			
**CD4/CD8 ratio**				
< 1.93	1			
≥ 1.93	0.31 (0.07–1.30)	0.108		0.111

ECOG PS, Eastern Cooperative Oncology Group performance status; mets, metastasis; ICI, immune checkpoint inhibitor; HR, hazard ratio; CI, confidence interval.

^a^Probability value by Cox regression model.

**Table 4:** Univariate and multivariate analyses of risk factors for progression free survival.

Variable	Univariate		Multivariate	
	HR (95% CI)	*p* value^a^	HR (95% CI)	*p* value^a^
Age	0.95 (0.89–1.02)	0.158		
Male gender	1.07 (0.34–3.37)	0.915		
Never smoker	1.31 (0.41–4.12)	0.650		
**ECOG PS**				
0–2	1			
3–4	6.00 (1.30–27.72)	0.022	6.78 (1.29–35.49)	0.024
Brain mets before ICI	1.02 (0.27–3.86)	0.980		
**PD-L1**				
< 1%	3.84 (0.77–19.33)	0.103		
1–49%	2.10 (0.34–12.91)	0.424		
≥ 50%	1			
**CD4/CD8 ratio**				
< 1.93	1			
≥ 1.93	0.27 (0.08–0.87)	0.028	0.25 (0.07–0.86)	0.027

ECOG PS, Eastern Cooperative Oncology Group performance status; mets, metastasis; ICI, immune checkpoint inhibitor; HR, hazard ratio; CI, confidence interval.

^a^Probability value by Cox regression model.

### Different types of pleural effusion

Representative images of different pleural effusion types were illustrated in online supplemental Figure 5. Type 1 patients within 2 weeks after ICI use developed pleural effusion with or without pericardial effusion (one patient also received pericardial drainage), and pigtail catheters were applied to two patients. All these patients presented with pleural effusion before ICI use and one of them was found to have malignant pleural effusion. Malignant cells were found in pleural effusion after the initial treatment but were then absent in the following serial thoracenteses. Type 2 patients usually developed pleural effusion one month after ICI use. Malignant pleural effusion was not documented before or after ICI use. Among type 3 patients, malignant pleural effusion persisted in 6 patients while disease progression to other organs was found in three patients.

Characteristics were compared between non-disease progression type (types 1 and 2) and disease progression type (type 3) (Table 1). In the non-disease progression group, 87.5% showed pleural effusion CD4/CD8 ratio ≥ 1.93, compared with 33.3% in the disease progression group (p = 0.036). The median OS in type 1 patients was not reached, whereas in type 2 was 24.8 months, and in type 3 was 2.6 months (Fig. 3B). The median PFS periods for patients with type 1, 2, and 3 effusion were 35.5, 30.2, and 1.4 months, respectively (Fig. 3D).

### Elevated pleural effusion B cell percentages in type 1 patients

Higher percentages of B cells were found in pleural effusions of patients without disease progression, especially in type 1 patients (Table 2). The optimal cutoff percentage of B cells was 6.09% in predicting OS, PFS, and pleural effusion types (online supplemental Figure 6). In a previous study, Nieto et al. reported CD20+ B lymphocytes account for 5.81% of all leukocytes in malignant pleural effusion^12^ so our finding of 6.09% is reasonable for defining an “elevated” B cell ratio. Of the 17 patients, 5 had elevated B cell ratios in their initial pleural effusion analyses, and 4 of them were with type 1 and type 2 effusions. Furthermore, high CD4/CD8 ratios also correlated with elevated B cell ratios (online supplemental Table 3). The B cell proportions of each patient are shown in online supplemental Figure 3D; while the proportions divided by type 1/2 and type 3 are shown in online supplemental Figure 3F.

### Higher IL-8 levels in patients with pleural effusion CD4/CD8 ratio < 1.93

Expression levels of cytokines in pleural effusion were shown in online supplemental Figure 7. IL-8 levels in the pleural effusion of patients with CD4/CD8 ratios < 1.93 were higher than those with ratio ≥ 1.93 (online supplemental Table 4). Expression levels of cytokines were however similar across effusion types (online supplemental Table 5).

## Discussion

We have here found different presentations of lung cancer patients developing pleural effusion after receiving ICI. Three effusion developmental patterns were identified. Type 1 patients developed massive effusion within one month after initiating ICI treatment, usually within 2 weeks. The first time cytological examinations of thoracentesis after treatment revealed positive for malignancy in all these patients. Their development of effusion could be interpreted as “pseudoprogression” because the cytological examinations turned negative in the serial thoracenteses afterwards. Fulminant effusion development was resolved within two months after ICI use.

Most researchers reported survival benefits of pseudoprogression markedly better than that of typical progression^13–16^. In our study, type 1 patients had longer PFS and OS than those of type 3 and type 2 patients. Some studies reported that the malignant pleural effusion present before anti-PD-1 treatment is associated with shorter PFS and OS^17^. In our study, if pseudoprogression occurred as type 1 pleural effusion, long-term survival could be achieved. Therefore, ICI should still be considered in patients with malignant pleural effusion.

Kolla et al. reported similar cases in which pseudoprogression was suspected after nivolumab administration^2^. One patient developing massive pleural effusion had frequent thoracenteses for 8 weeks after nivolumab use. The cytological examination from pleural effusion was positive for malignancy. Nivolumab continued and there was a complete response. No more drainage was recorded after the first two months of therapy. That case shared a similar clinical presentation with our type 1 patients and may be categorized as “type 1” pleural effusion.

Type 2 pleural effusion developed one month after ICI treatment had begun. The time from treatment to the first thoracentesis was as long as 25 months (case no.8). It is not surprising that the occurrence of irAEs was delayed since Nigro et al. reported earlier that late-irAEs (after 12 months) are common (incidence 30.3%) in long responders to ICIs^18^. The cytology of pleural effusion was never documented positive before or after ICI use so this type was not categorized as pseudoprogression. Thoracentesis was usually infrequent. The exception was case no.7. He presented with typical type 2 pleural effusion in the first year after ICI treatment. However, disease progression was noted and the cytological result of pleural effusion turned positive 14 months later. Subsequent infection of massive pleural effusion after disease progression developed and pigtail was therefore inserted.

We identified those with CD4/CD8 ratios of pleural effusion ≥ 1.93 were well predicted for their survival. Though the initial CD4/CD8 ratio of patient no.1 (type 1) was only 1.89, serial pleural effusion CD4/CD8 ratios examined showed progressively elevated ratios, with the highest reaching 13.8. It is worth noting that the B cell ratio of pleural effusion was also elevated from 0.4 to 22.1% in serial analyses (Fig. 2). For such patients presented with the typical type 1 or type 2 pleural effusions with CD4/CD8 ratios < 1.93, serial follow-up is recommended, because the elevation of the ratio may indicate a good response to the ICI treatment. In type 1 patients, elevated B cell percentage is the feature distinguishing them from types 2 and 3 patients. However, B cell percentage varies among patients (0.3–34%) which was patient-specific and any increase in B cells should be made by comparison with a pre-treatment sample. Therefore, we should interpret the proportions with caution.

Three type 3 patients had initial pleural effusion CD4/CD8 ratios ≥ 1.93. Patient no.11 and 12 showed partial responses at the primary lesion, but the development of new lesions was noted during follow-up. No further treatment was given after disease progression due to poor performance status. This challenged the interpretation of OS. Patient no.17 developed pleural effusion 19 months after starting durvalumab medication, and CD4/CD8 and B cell ratios then increased, while cytological results were positive. Nevertheless, the PFS of this patient went up to 18.4 months. Infrequent thoracenteses were performed and there’s no disease progression to the other organs beyond the pleura. Longer follow-ups are desirable as the clinical presentation was different from other type 3 patients.

Infiltration of inflammatory cells with CD4+ predominant may contribute to elevated CD4/CD8 ratio in the pleural effusion. Scherpereel et al. evaluated T cell populations in patients with pleural effusion. Their blood CD4/CD8 ratios were 1.6. In healthy subjects, the ratio in pleural fluid is 0.59, compared with higher ratios of 3.8 in patients with pleural metastasis^19^. Aguiar et al. found CD4/CD8 ratios were similarly higher in malignant pleural effusion than in the peripheral blood (i.e., 3.6 vs 1.4)^20^. Nieto et al. reported that in patients after diagnosing malignant pleural effusion, their lymphocytes count in the pleural effusion is positively correlated with survival. CXCL10 helps attract lymphocytes in malignant effusion^12^. Accordingly, in patients with malignant pleural effusion, the CD4/CD8 ratio of which is higher than the peripheral blood. This may be a defensive mechanism against cancer, and ICI likely reinforces the mechanism.

Regarding irAEs, cytokines or chemokines in response to ICIs have been studied. Khan et al. reported irAEs patients have initially low levels of CXCL9, 10, 11, and 19, but levels of CXCL 9, and 10 remarkably increase after treatment compared with those patients without irAEs^21^. Lim et al. found elevations of 11 cytokines in patients with severe irAEs, and even introduced a cytokine toxicity score^22^. IL-17 and IL-6 levels were reported as biomarkers in predicting irAEs^23,24^. In our study, we found IL-8 levels in patients with pleural effusion CD4/CD8 ratio < 1.93 were higher than those with ratio ≥ 1.93. IL-8, a chemokine produced by cancer cells, could play a role in the cancer microenvironment. Higher IL-8 levels are correlated with poor prognosis^25^. Only one patient from the type 1 group has a higher level of IL-17. We also examined several other cytokines including IL-1, IL-2, IL-4, IL-6, IL-12p70, INF-γ, and TNF-α. However, the levels of these cytokines are either under detection limit or demonstrate no significant difference among the three types of patients.

There were several limitations of our study. First, its sample size was small and was conducted retrospectively in a single medical center. Second, not all patients had their CD4/CD8 ratios determined at the initial thoracentesis. Also, their CD4/CD8 ratios were not determined before ICI treatment nor their ratio in the peripheral blood. Third, 11 of 17 patients received both chemotherapy and ICI, presenting a confounder on response evaluation. However, no patient was lost during follow up and all required clinical information was collected. We are the first to report two distinct types of pleural effusions after ICI use. These two types of patients both had relatively good prognoses. Our study is also the first to use the CD4/CD8 ratio in pleural effusion to predict patient survival after ICI use.

In conclusion, besides pleural effusion due to disease progression (type 3), two distinct effusion types were identified after ICI use: type 1, rapid (develop < 1 month) and massive and type 2, slow (develop ≥ 1 month), and relative indolent. Both types showed better overall and progression-free survival than type 3. Type 1 could be interpreted as pseudoprogression of malignant pleural effusion. CD4/CD8 ratio ≥ 1.93 in pleural effusion after ICI use is a good predicting factor in PFS. In most patients of types 1 and 2, their CD4/CD8 ratios ≥ 1.93 in pleural effusion. In those patients presented with typical type 1 or type 2 pleural effusion but with CD4/CD8 ratios < 1.93, serial follow-up is recommended because elevating ratio may indicate a good response to ICI.

## Supplementary Information

Supplementary Information.
