# Supplementary material for: Higher CD4/CD8 ratio of pleural effusion predicts better survival for lung cancer patients receiving immune checkpoint inhibitors
Source: Sci Rep. 2021 Apr 30;11:9381. doi: 10.1038/s41598-021-89043-4 (PMC8087817; doi:10.1038/s41598-021-89043-4)
Supplement: Supplementary file 1 — Supplementary Information. [file 41598_2021_89043_MOESM1_ESM.docx]

**Supplementary information**

Higher CD4/CD8 ratio of pleural effusion predicts better survival for lung cancer patients receiving immune checkpoint inhibitors

**Authors:**

Po-Hsin Lee, Tsung-Ying Yang, Kun-Chieh Chen, Yen-Hsiang Huang, Jeng-Sen Tseng, Kuo-Hsuan Hsu, Yu-Chen Wu, Ko-Jiunn Liu*, Gee-Chen Chang*

***Gee-Chen Chang**

Division of Pulmonary Medicine, Department of Internal Medicine, Chung Shan

Medical University Hospital, No. 110, Sec. 1, Jianguo N. Rd., South Dist., Taichung

City 402, Taiwan, R.O.C.

E-mail: geechen@gmail.com

***[Ko-Jiunn Liu](https://pubmed.ncbi.nlm.nih.gov/?term=Liu+KJ&cauthor_id=31963522)**

National Institute of Cancer Research, National Health Research Institutes, 2F, No.367, Sheng-Li Road, Tainan 70456, Taiwan

E-mail: kojiunn@nhri.edu.tw

**Supplemental Table 1.** Details of equipment and antibody

| Ficoll‐Paque | Amersham Pharmacia Biotech, Uppsala, Sweden |
| --- | --- |
| Flow cytometer | FACSVerse, BD Biosciences, Franklin Lakes, NJ |
| Phycoerythrin (PE)‐anti-CD3 | BD PharMingen, Bedford, MA (Clone HIT3) |
| PerCP‐anti-CD4 | BioLengend, Burlingame, CA (Clone OKT4) |
| FITC‐anti-CD8 | Immunotech, Marseille Cedex, France (Clone B9.11) |
| PE-anti-CD19 | BD PharMingen (Clone HIB19) |

**Supplemental Table 2.** Detection ranges of cytokines in pleural effusion by ELISA

| Cytokine | Range |
| --- | --- |
| IFN-γ | 4.7–300 pg/ml |
| TNF-α | 15.6–1000 pg/ml |
| IL-1 | 7.9–500 pg/ml |
| IL-2 | 15.6–1000 pg/ml |
| IL-4 | 15.6–1000 pg/ml |
| IL-6 | 31.2–2000 pg/ml |
| IL-8 | 31.2–2000 pg/ml |
| IL-10 | 15.6–1000 pg/ml |
| IL-12p70 | 7.9–500 pg/ml |
| IL-13 | 4–500 pg/ml |
| IL-17 | 15.6–1000pg/ml |

**Supplemental Table 3.** Distribution of pleural effusion types, PD-L1 expression, and B cell ratio among high and low CD4/CD8 ratio

|  | All | CD4/CD8 >=1.93 | CD4/CD8 <1.93 | *p* Value^a)^ |
| --- | --- | --- | --- | --- |
|  | (N=17) | (N=10) | (N=7) |  |
| Pleural effusion types, N (%) |  |  |  | 0.036 |
| Type 1 + Type 2 | 8 (47.1) | 7 (70) | 1 (14.3) |  |
| Type 3 | 9 (52.9) | 3 (30) | 6 (85.7) |  |
| PD-L1, N (%) |  |  |  | 0.012 |
| <1% | 6 (35.3) | 1 (10) | 5 (71.4) |  |
| 1-49% | 4 (23.5) | 3 (30) | 1 (14.3) |  |
| >=50% | 5 (29.4) | 5 (50) | 0 (0) |  |
| N/A | 2 (11.8) | 1 (10) | 1 (14.3) |  |
| B cell ratio, N (%) |  |  |  | 0.044 |
| >= 6.09 | 5 (29.4) | 5 (50) | 0 (0) |  |
| < 6.09 | 12 (70.6) | 5 (50) | 7 (100) |  |
| a)Probability value by Fisher's exact test. | | |  |  |

**Supplemental Table 4.** Expression levels of cytokines in pleural effusion of patients with CD4/CD8 ratio >=1.93 versus ratio < 1.93

|  | CD4/CD8 ratio>=1.93 | CD4/CD8 ratio<1.93 | *p* Value |
| --- | --- | --- | --- |
| IL-6 (mean+/-SD) | 4971+/-5774 | 5110+/-4521 | 0.962 |
| IL-8 (mean+/-SD) | 376+/-609 | 1707+/-1141 | 0.019 |
| IL-10 (mean+/-SD) | 64+/-63 | 31+/-26 | 0.230 |

**Supplemental Table 5.** Expression levels of cytokines in pleural effusion of patients with type 1 + type 2 versus type 3

|  | Type 1 + Type 2 | Type 3 | *p* Value |
| --- | --- | --- | --- |
| IL-6 (mean+/-SD) | 5468+/-6438 | 4637+/-4026 | 0.963 |
| IL-8 (mean+/-SD) | 681+/-804 | 1140+/-1275 | 0.743 |
| IL-10 (mean+/-SD) | 56+/-64 | 45+/-44 | 0.743 |


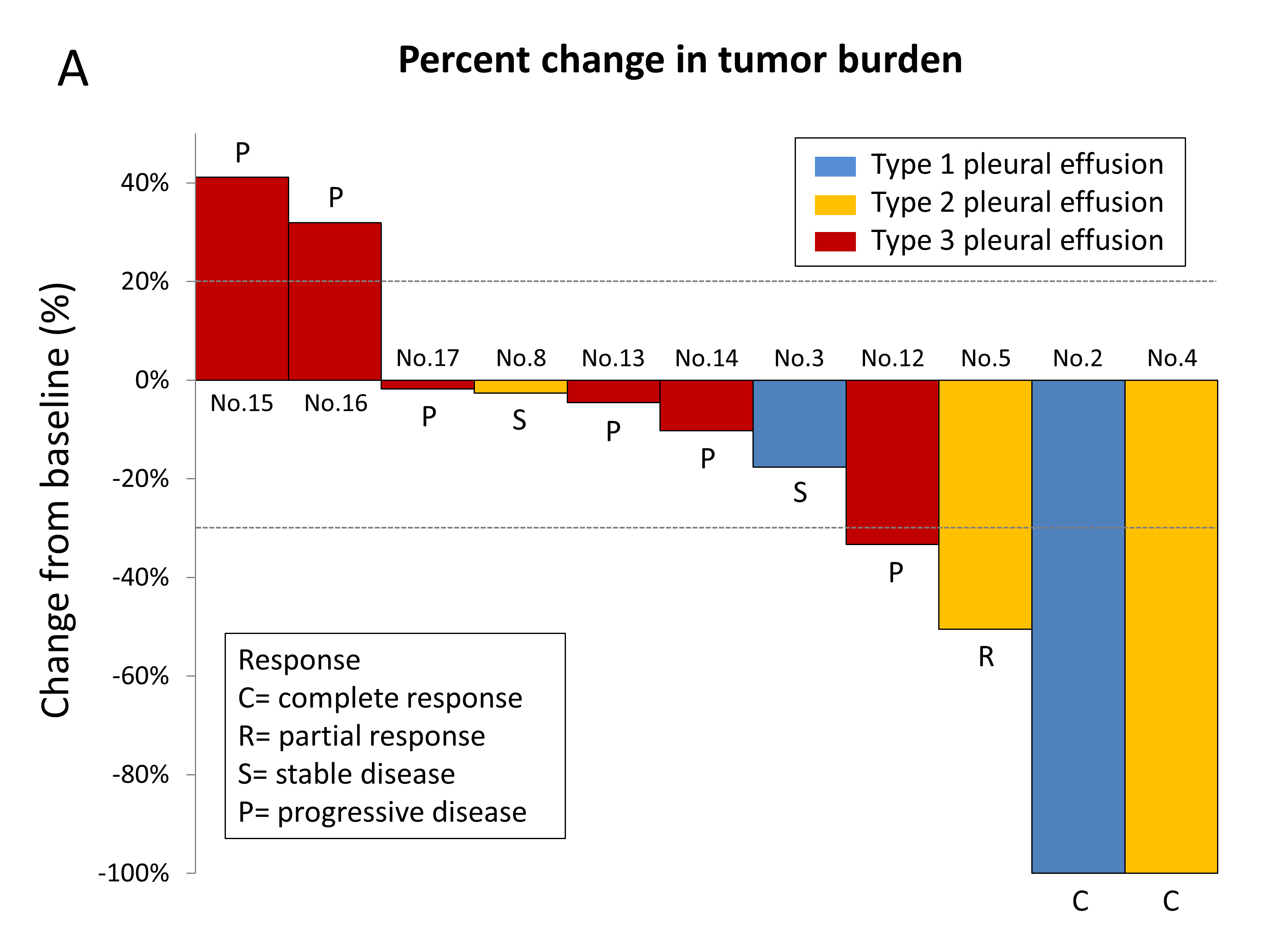

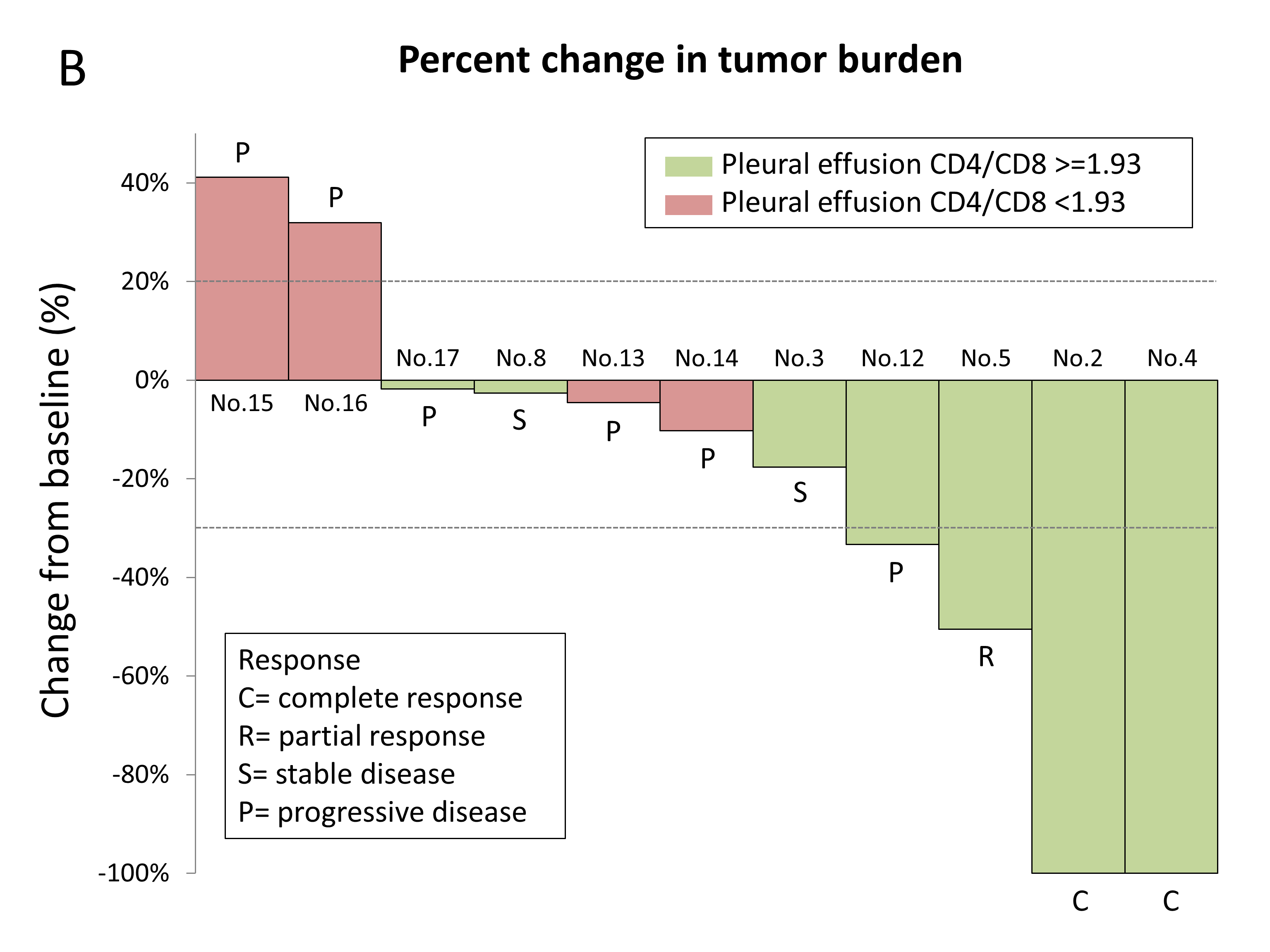


**Supplemental Figure 1.** Radiological change for each patient with measurable lesion compared with baseline diameter according to (A) pleural effusion pattern, or (B) pleural effusion CD4/CD8 ratio.


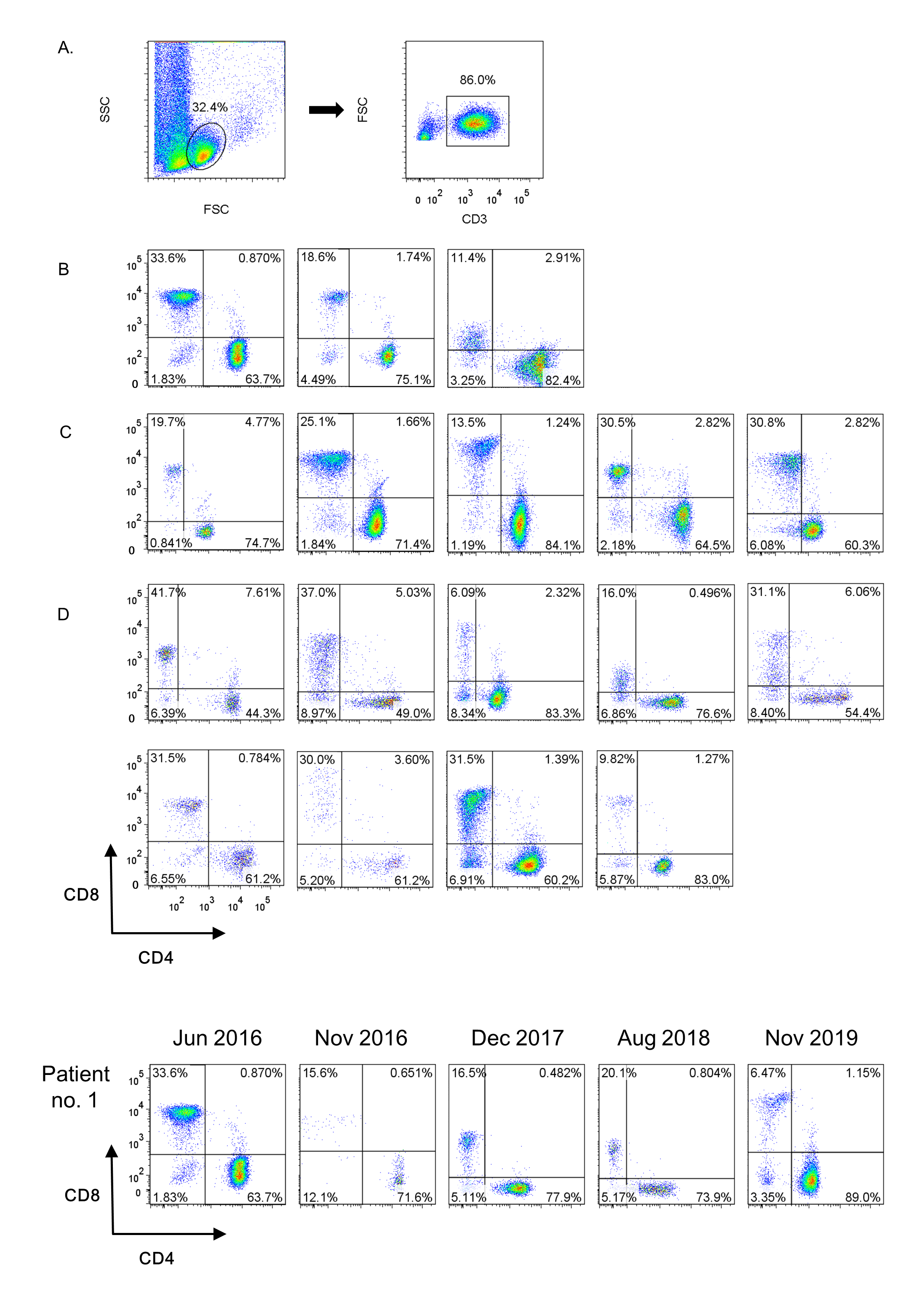


**Supplemental Figure 2.** Cell surface expressions of CD4 and CD8 on T cells in the pleural effusion of patients.

Mononuclear cells in the pleural effusion were collected by density gradient centrifugation. Cells were first gated based on forward scatter channel and side scatter channel to select lymphocytes (A, left panel). For T cell subset study, cells were stained with PE‐anti-CD3, PerCP‐anti-CD4, and FITC‐anti-CD8. Cells expressing CD3 were gated (A, right panel) for the analyses of CD4 and CD8 expression. B: cells from three type 1 patients; C: cells from five type 2 patients; D: cells from nine type 3 patients. Patient no.1: serial cells from patient number 1.


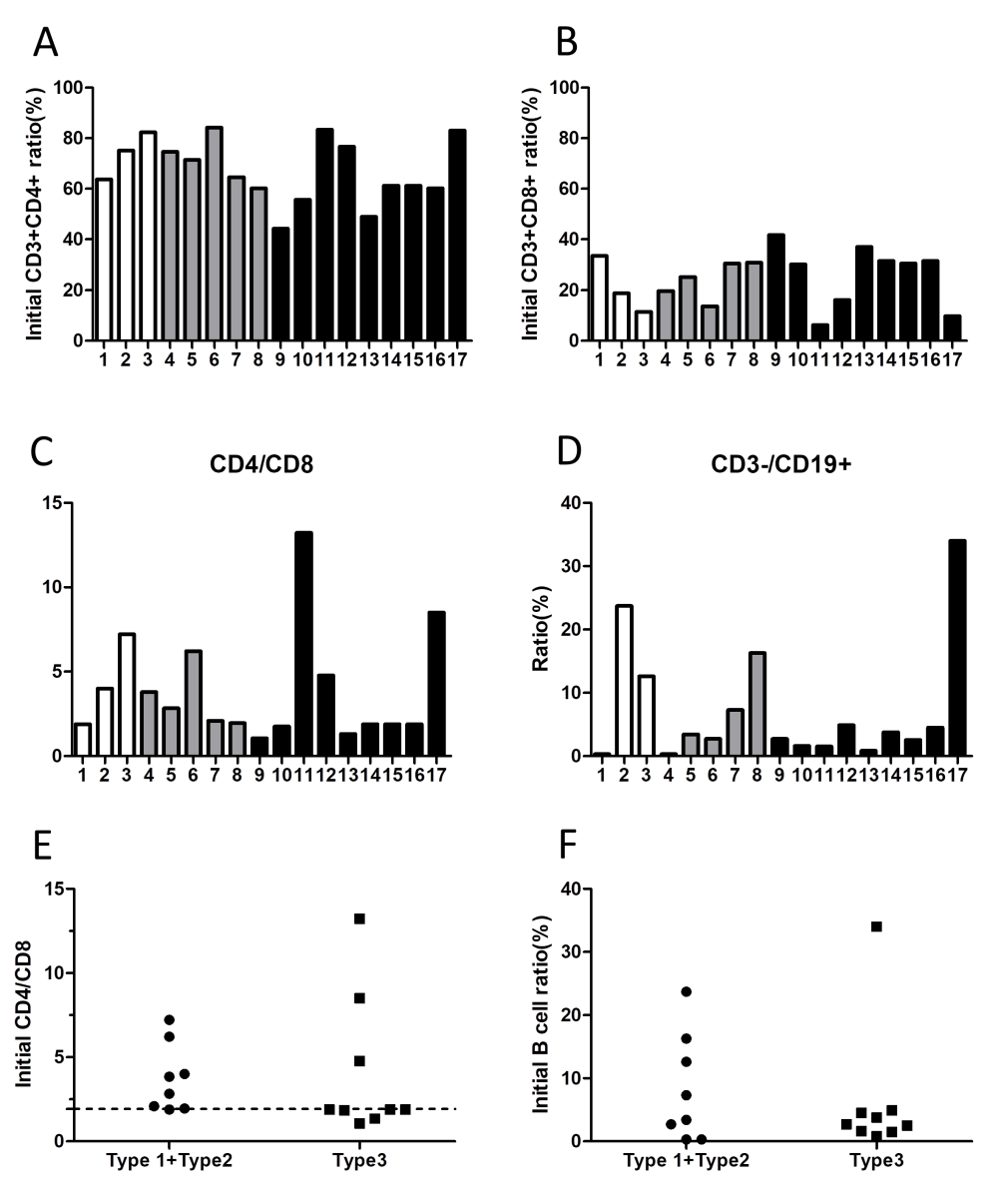


**Supplemental Figure 3.** The CD4, CD8 ratios and B cell proportions of each patient and pleural effusion subtype.
(A) The initial CD4 ratios and (B) CD8 ratios as a proportion of cells expressing CD3 of each patient. White bars: type 1 patients; Grey bars: type 2 patients; Black bars: type 3 patients. (C) The initial CD4/CD8 ratios and (D) B cell proportions of each patient. (E) The CD4/CD8 ratios divided by type 1/2 and type 3 pleural effusion. The dotted line indicate the cutoff threshold of 1.93. (F) The B cell proportions divided by type 1/2 and type 3 pleural effusion.


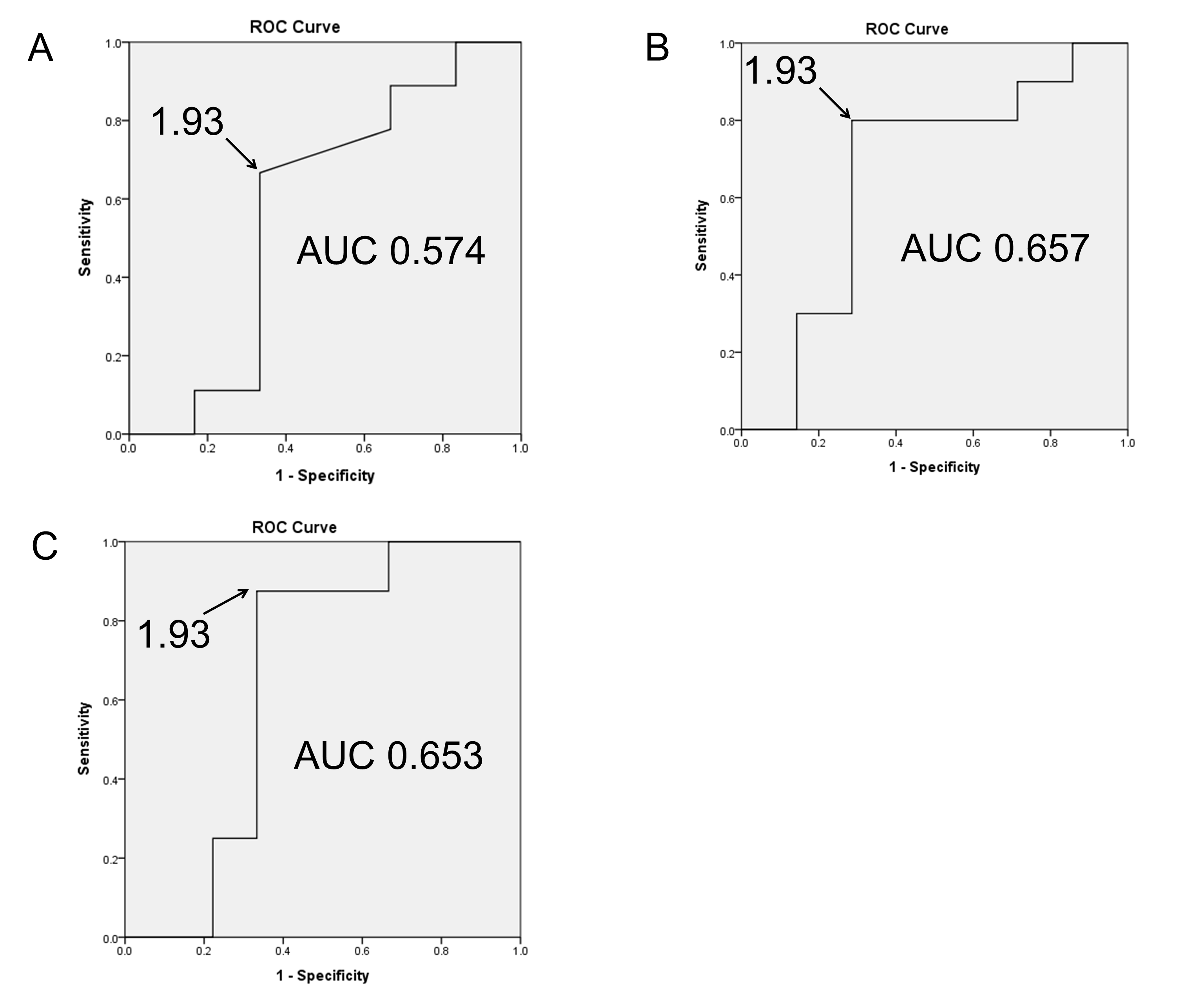


**Supplemental Figure 4.** Receiver operating curves of CD4/CD8 ratio.

(A) CD4/CD8 ratio in predicting overall survival. (B) CD4/CD8 ratio in predicting progression free survival. (C) CD4/CD8 ratio in predicting pleural effusion types.


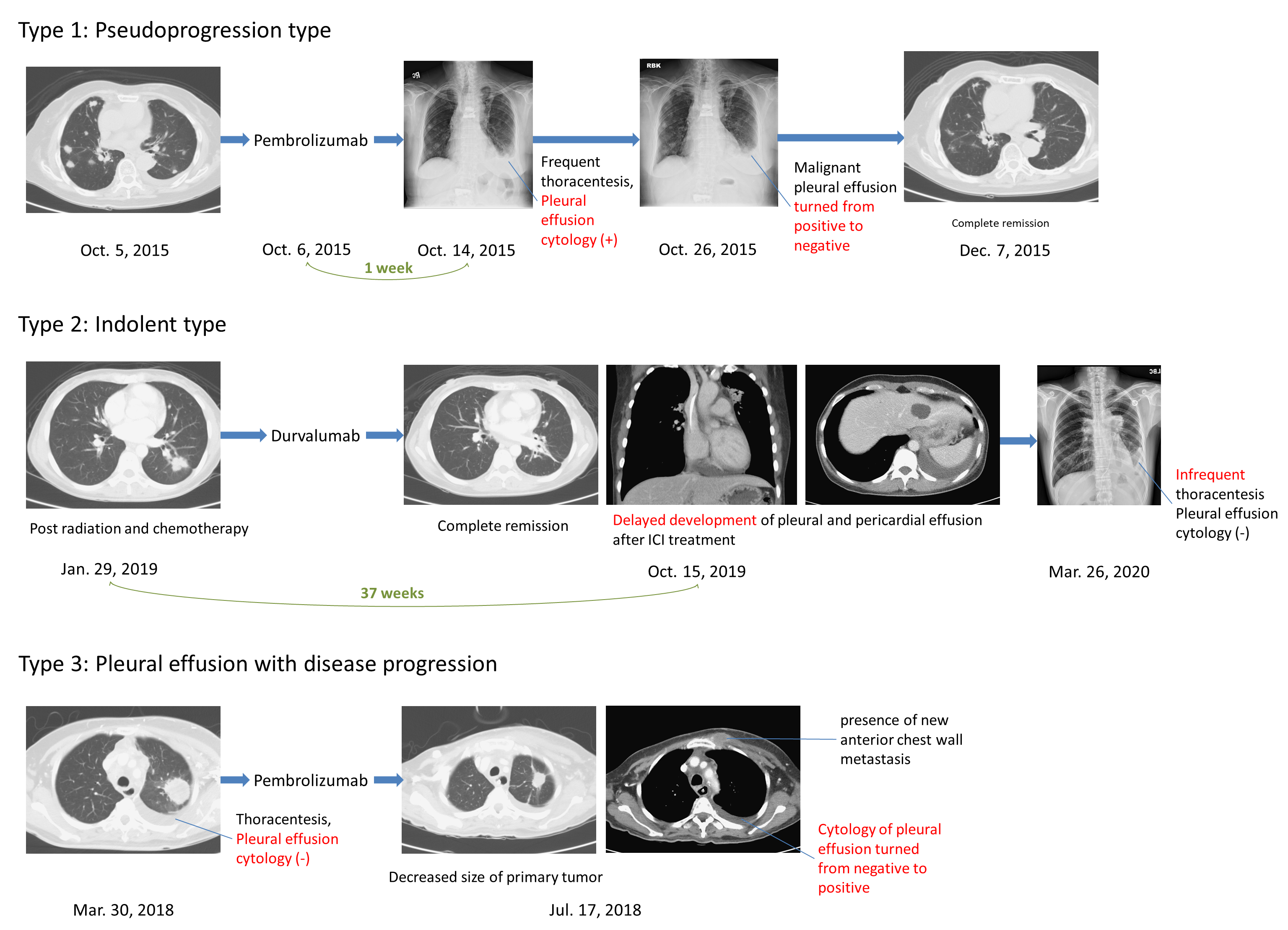


**Supplemental Figure 5. Representative images of different pleural effusion types**


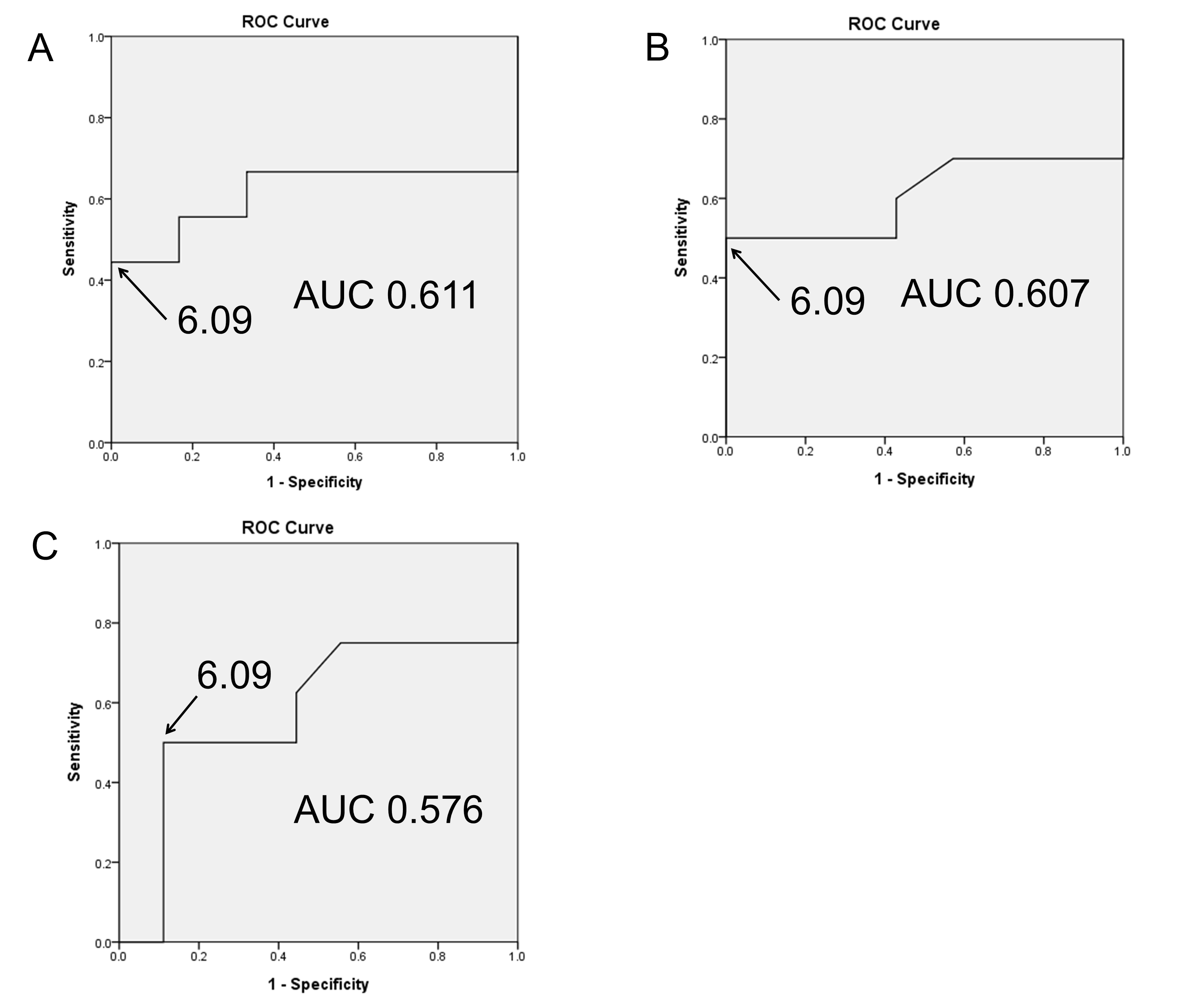


**Supplemental Figure 6.** Receiver operating curves of B cell ratio.

(A) B cell ratio in predicting overall survival. (B) B cell ratio in predicting progression free survival. (C) B cell ratio in predicting pleural effusion types.


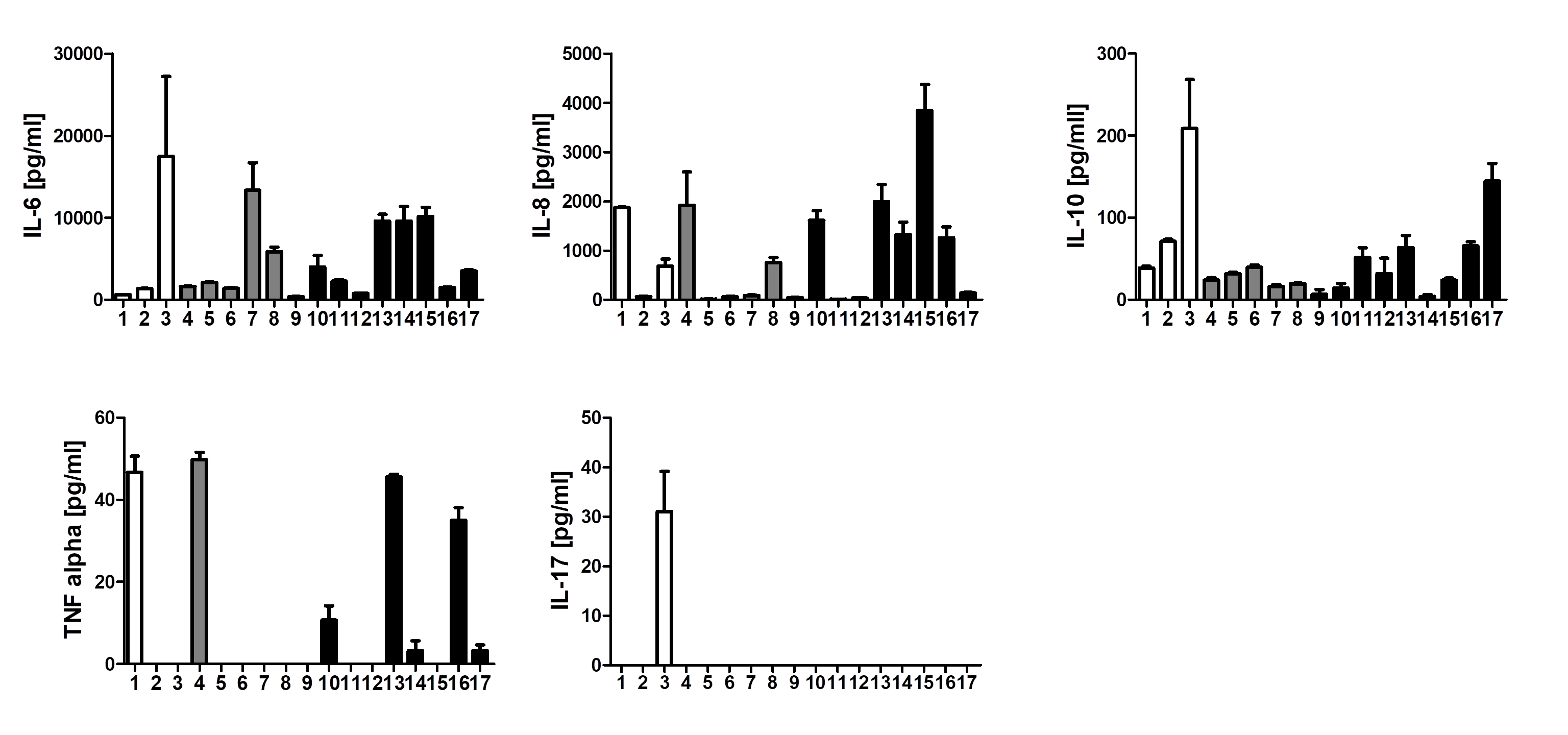


**Supplemental Figure 7.** Expression levels of selected cytokines in pleural effusion of patients

Pleural effusion from patients was centrifuged to remove cells and debris. Levels of various cytokines in the pleural effusion were detected by ELISA as described in the Methods. White bars: type 1 patients; Grey bars: type 2 patients; Black bars: type 3 patients.
